# Phase II study of the oral selective inhibitor of nuclear export (SINE) KPT-335 (verdinexor) in dogs with lymphoma

**DOI:** 10.1186/s12917-018-1587-9

**Published:** 2018-08-24

**Authors:** Abbey R. Sadowski, Heather L. Gardner, Antonella Borgatti, Heather Wilson, David M. Vail, Joshua Lachowicz, Christina Manley, Avenelle Turner, Mary K. Klein, Angharad Waite, Alexandra Sahora, Cheryl A. London

**Affiliations:** 1 Cummings School, Tufts University, Foster Hospital for Small Animals, 200 Westboro Rd, N. Grafton, MA 01536 USA; 2 Sackler School of Graduate Biomedical Sciences, Tufts University, Boston, MA USA; 3 College of Veterinary Medicine, University of Minnesota, St. Paul, MN USA; 4 College of Veterinary Medicine, Texas A&M University, College Station, TX USA; 5 Department of Medical Sciences, School of Veterinary Medicine, University of Wisconsin-Madison, Madison, WI USA; 6 NYC Veterinary Specialists/Blue Pearl Veterinary Specialists, New York, NY USA; 7 The Oncology Service, The LifeCentre, Leesburg, VA USA; 8 Veterinary Cancer Group, Culver City, CA USA; 9 Southern Arizona Veterinary Specialty and Emergency Center, Tucson, AZ USA; 10 The Oncology Service, Dogwood Veterinary Emergency and Specialty Center, Richmond, VA USA; 11 The Oncology Service, Friendship Hospital for Animals, Washington DC, USA; 12 Department of Veterinary Biosciences, College of Veterinary Medicine, The Ohio State University, Columbus, OH USA

**Keywords:** Non-Hodgkin lymphoma, Nuclear export, Clinical trial, Anti-tumor agent

## Abstract

**Background:**

Chemotherapeutic options for the treatment of canine lymphoma have not changed in several decades necessitating the identification of new therapeutics to improve patient outcome. KPT-335 (verdinexor) is a novel orally bioavailable selective inhibitor of nuclear export (SINE) that exhibited anti-tumor activity against non-Hodgkin lymphoma in a prior phase I study. The objective of this phase II study was to expand upon the initial findings and assess the activity and safety in a larger population of dogs with lymphoma.

**Results:**

Fifty-eight dogs with naïve or progressive B-cell and T-cell lymphoma were enrolled in this clinical trial. KPT-335 was administered orally in one of three dosing groups, based on the previously established biologically active dose of 1.5 mg/kg three times weekly. Treatment with single-agent, orally administered KPT-335 resulted in an objective response rate (ORR) of 37%, of which dogs with T-cell lymphoma had an ORR of 71%. KPT-335 was well tolerated in all dose groups with grade 1–2 anorexia being the most common adverse event. Anorexia was responsive to symptomatic and supportive medications, including prednisone.

**Conclusions:**

These data demonstrate that KPT-335 has biologic activity in canine lymphoma, and support continued evaluation of SINE compounds such as KPT-335 in combination with standard chemotherapeutics in canine lymphoma.

**Electronic supplementary material:**

The online version of this article (10.1186/s12917-018-1587-9) contains supplementary material, which is available to authorized users.

## Background

The transport of specific proteins between the nucleus to the cytoplasm is critical for the normal function of tumor suppressor proteins (TSP) and growth regulatory proteins (GRP). Neoplastic cells utilize the process of nucleo-cytoplasmic transport to export known TSP and GRP outside of the nucleus, inactivating these pathways and overcoming the normal cell cycle and genomic instability checkpoints [1, 2]. Nuclear export of proteins depends on the activity of transport proteins called exportins. Exportin-1, also known as XPO1, is the main mediator of nuclear export. XPO1 inhibition forces the nuclear retention of key TSP and GRP such as p53, p21, pRB, FOXO and NF-κB [3, 4].

XPO1 is overexpressed in many hematologic and nonhematologic malignancies in humans and is associated with a poor prognosis in aggressive diseases [3]. Several small molecule inhibitors targeting XPO1 have been investigated, including KPT-251, KPT-276 and KPT-330 [5]. A phase I study of orally administered KPT-330 showed safety and feasibility of long-term treatment in a variety of patients with advanced solid tumors. Out of 157 patients, 27 patients (17%) achieved stable disease for four months or longer [6]. Given the safety profile and cytotoxic effects of KPT-330 on rapidly proliferating leukemia cells in animal models and patient samples, several studies have investigated the efficacy and safety of KPT-330 in refractory or relapsed acute myeloid leukemia and non-Hodgkin’s lymphoma [7, 8]. KPT-330 was efficacious in refractory or relapsed AML patients, with a greater than 50% decrease in bone marrow blasts and significant improvement in overall survival in responders vs. nonresponders (9.7 months vs 2.7 months) [9]. Similarly, a phase I trial of people with relapsed or refractory lymphoma showed an objective response rate of 31% (22/71 patients) [10]. Therefore, XPO1 is an attractive target for patients with aggressive hematologic cancers.

KPT-335 is an orally bioavailable selective inhibitor of nuclear export (SINE) that transiently binds to XPO1 in a slowly reversible manner [3, 4]. KPT-335 was recently shown to be safe and biologically active in a phase I study of dogs with spontaneous cancer [11]. The maximum tolerated dose was found to be 1.75 mg/kg administered twice weekly, with biologic activity observed at 1 mg/kg. Clinical benefit was seen in 9/14 dogs with Non-Hodgkin’s lymphoma leading to a dose expansion study of 6 dogs given KPT-335 at 1.5 mg/kg on Monday/Wednesday/Friday. Clinical benefit was demonstrated in 4 out of the 6 dogs [11].

The primary objective of this phase II study was to build upon the phase I study findings, and describe the safety and anti-tumor activity of oral KPT-335 in dogs with naïve lymphoma, or after a single relapse. These data will provide information on the best use of SINE compounds in future clinical studies.

## Methods

### Clinical trial eligibility

This clinical trial was approved by the Animal Clinical Investigation (ACI) Animal Care and Use Committee (ACUC); IACUC or equivalent approval was obtained at all participating study sites. Written informed consent was obtained from each pet owner prior to study entry. KPT-335 was administered to dogs with newly diagnosed or first relapse B-cell or T-cell non-Hodgkin’s lymphoma (NHL). Dogs were administered KPT-335 orally until disease progression or intolerance to the drug. To be eligible for the study, each dog must have had a cytologic or histologic diagnosis of B or T cell lymphoma and met all the inclusion criteria and none of the exclusion criteria. Eligibility criteria included: at least 1 year of age, at least one measurable peripherally located tumor-infiltrated lymph node, adequate organ function, dogs with naïve lymphoma or experiencing first relapse on study entry, completion of any prior chemotherapy at least 14 days prior to study entry and no evidence of central nervous system involvement or concomitant serious systemic disorder as determined by the supervising clinician.

### KPT-335 formulation

KPT-335 was prepared as whole tablets of 2.5, 10 and 50 mg strengths of active ingredient (KABS – St-Hubert Quebec, Canada), using pharmaceutical grade excipients.

### Study design

This was a phase II open-label field study of the safety and anti-tumor activity of KPT-335 in client owned dogs with lymphoma. Analysis of dogs for tumor response was performed by caliper measurement of the longest diameter of a maximum of five lesions. Tumor response was determined using the VCOG peripheral nodal lymphoma response criteria [12]. A partial response (PR) was identified if the target lesions demonstrated at least a 30% decrease in the mean sum of the longest diameter. A complete response (CR) was defined as complete disappearance of disease, evaluated by caliper measurements. Progressive disease was defined by at least a 20% increase in the mean sum of the longest diameter, using the smallest sum since initiation of the clinical trial as a reference, or any new lesion. Dogs were assessed as having stable disease (SD) if they did not meet the criteria for PR or PD. Assessment of hematologic and biochemical toxicities was performed at screening and every 7 days until day 28, and then every 2 weeks until study discontinuation. Time to progression (TTP) was defined as the period of time from the first date of treatment to the date that the dog developed progressive disease because of clinical or radiographic progressive disease or death from any cause, including euthanasia. Duration of Response (DOR) was defined as the period of time between the first of two evaluations demonstrating an objective response until the date of progression or death. Disease Control Rate (DCR) was defined as the number of patients with confirmed complete or partial responses, OR stable disease, as a percentage of all patients treated with KPT-335 in this protocol. Objective Response Rate (ORR) was defined as the number of patients with confirmed complete or partial responses as a percentage of all evaluable patients treated with KPT-335 in this protocol.

Dogs were initially treated with 1.5 mg/kg KPT-335 orally three times weekly (Monday, Wednesday, Friday) (Group 1) based on previous data showing safety and clinical benefit in dogs at this dose [11]. Two additional dosing groups were implemented at a decreased dose of 1.25 mg/kg PO three times weekly (Group 2) and 1.25 mg/kg PO twice weekly for 2 weeks then 1.5 mg/kg PO twice weekly thereafter (Group 3).

### Treatment administration and adverse events

KPT-335 was administered orally using whole tablets immediately following feeding. Adverse events (AEs) were reported using the Veterinary Cooperative Oncology Group- common terminology criteria for adverse events, VCOG-CTCAE v 1.1 [12]. AEs were defined as any expected or unexpected grade 1, 2, or 3 toxicity, whereas a Serious Adverse Event (SAE) was any expected or unexpected grade 4 or 5 toxicity. Dose reductions (0.25 mg/kg) or temporary drug discontinuation not extending beyond 7 days were permitted in dogs experiencing AEs.

### Concomitant medications

No concomitant anti-neoplastic therapy (chemotherapy, immunotherapy, radiation therapy) was permitted during the clinical trial. Low-dose prednisone (0.5–1.0 mg/kg/day) was permitted if necessary to treat AEs, and was also permitted in dogs already receiving prednisone that demonstrated PD at study entry. Supportive care medications were administered as clinically needed to treat drug-related toxicities. These included metoclopramide, ondansetron, maropitant, famotidine, ranitidine, omeprazole, metronidazole and loperamide. Other supportive medications permitted included butorphanol, tramadol, fentanyl, diphenhydramine, s-adenosylmethionine and mirtazapine.

### Pharmacokinetics analysis

Complete pharmacokinetic (PK) sampling was performed on 8 dogs. Blood samples were collected at 0, 1, 2, 4, 6, 8, 12 and 24 h on day 14, with the exception of one dog who had PK sampling on day 21. A minimum of 2 ml of whole blood was collected in an EDTA tube. The sample was centrifuged at room temperature (3000 rpm for 10 min) and cells were separated from the plasma. Plasma was aliquoted into cryovials and stored in liquid nitrogen within 20 min of collection. Plasma samples were analyzed for KPT-335 using a validated LC/MS/MS method. Parameters evaluated included peak concentration (Cmax), time of observed maximum concentration (Tmax), terminal half-life t_1/2_, Area under the curve (AUC) of analyte vs. time curve from time zero to infinity (AUC_inf_), AUC of analyte vs. time curve from time zero to the time of the last measurable concentration (AUC_last_).

## Results

### Patient demographics

A total of 58 dogs were enrolled and received at least one dose of KPT-355 across 10 study sites. Thirty-five dogs with naïve lymphoma and 23 dogs at the time of their first relapse were enrolled in this clinical trial. Of the 35 naïve cases, 28 dogs had B-cell lymphoma and 7 dogs had T-cell lymphoma. The majority of dogs were classified as having stage III or IV lymphoma (*n* = 31) based on staging tests including physical examination, complete blood count, serum biochemical panel and thoracic radiographs. The mean age of chemotherapy-naïve dogs was 7 years (median 7; range 3–12) and mean weight was 31 kg (median 31; range 6–85). The number of male dogs (*n* = 20, 2 intact) outnumbered female dogs (*n* = 15, all spayed). Of the 23 relapsed lymphoma patients, 14 had B-cell lymphoma and 7 had T-cell lymphoma. Two relapsed lymphoma patients did not have immunophenotyping. The mean weight and age of relapsed dogs was 27 kg (Median 28; range 5–52) and 8 years (Median 7; range 3–13), respectively. As with naïve lymphoma, males (*n* = 16, 2 intact) outnumbered females (*n* = 7, all spayed). Patient demographic data is summarized in Table 1. Of the 58 dogs enrolled in the study, 54 dogs were considered evaluable for responses. Dogs that were considered evaluable for response were dogs that had one baseline assessment and at least one lesion assessment two weeks following the start of therapy. All fifty-eight dogs were evaluated for AEs related to drug administration.

**Table 1:** Patient Demographics

	All (*N* = 58)	Group 1 (*N* = 13)	Group 2 (*N* = 35)	Group 3 (*N* = 10)
Median Weight (kg)	30.1	25.2	30.3	33.3
Range (Min, Max)	5, 85	6, 53	6, 85	5, 52
Median Age (yr)	7	7	7	7
Range (Min, Max)	3, 12	4, 12	3, 12	3, 13
Sex				
Female	22	7	11	2
Male	36	6	23	7
Stage				
II	1	0	1	0
III	36	7	21	6
IV	18	5	10	3
V	3	1	2	0
Immunophenotype				
B-cell (Naïve)	28	9	19	0
B-cell (Relapsed)	14	2	6	6
T-cell (Naïve)	7	1	6	0
T-cell (Relapsed)	7	1	3	3
Not Determined	2	0	1	1
Corticosteroid Use				
Prior prednisone	15	1	8	6
Prednisone use after enrollment	25	7	15	3
No prednisone use	18	5	11	1

### Response to therapy

For all dogs, the ORR was 37% (20/54) in the evaluable patient population. The median DOR was 18 days (range: 7–152), median TTP was 29 days (range: 7–244) and median study duration was 36 days (range: 7–273). Twenty dogs (36%) remained on study for at least 56 days. Of the evaluable patients with naïve lymphoma, the DCR was 33%, ORR 39%, and median DOR was 34 days. The DCR was 29%, ORR of 38% and median DOR was 19 days for dogs with relapsed NHL.

Time to Tumor progression was assessed for all dose groups. The median TTP was 43 days for naïve lymphoma patients (*n* = 33). The median TTP for relapse lymphoma patients was 24 days (*n* = 21). Table 2 illustrates the TTP data for each dosing group and the distinct immunophenotypes, with and without corticosteroid usage. Of note is the median TTP for first relapse dogs with T-cell lymphoma, with a TTP of 43 days (*n* = 7). The median TTP for dogs receiving corticosteroids on or after Day 0 was 73 days compared to dogs on corticosteroids prior to enrollment or not on corticosteroids (24 and 22 days, respectively).

**Table 2:** Patient Response Evaluation

	All (*N* = 54)	Group 1 (*N* = 13)	Group 2 (*N* = 31)	Group 3 (*N* = 10)
TTP (days)				
Naïve B-cell	44	71	35	–
Naïve T-cell	31	31	32.5	–
Relapsed B-cell	24	19	23	85
Relapsed T-cell	43	43	73	24
Objective Response (n)				
Naïve B-cell	8	4	4	–
Naïve T-cell	5	1	4	–
Relapsed B-cell	4	1	1	2
Relapsed T-cell	4	1	2	1

### Pharmacokinetics

Seven dogs underwent full PK analysis to determine KPT-335 plasma levels over a 24 h time period post KPT-335 administration on day 14. The dogs received KPT-335 at 1.5 mg/kg (*n* = 4) or 1.25 mg/kg (*n* = 4) on MWF dosing schedule. For all dogs, the mean Cmax was 278 ng/ml with a mean AUC of 1970.6 ng*t/ml, Tmax of 5.3 h and T_1/2_ of 5 h. The mean Cmax and AUC for dogs receiving 1.5 mg/kg were slightly higher than dogs receiving 1.25 mg/kg (312 vs. 244.8 mg/ml and 2346.8 vs. 1576.5 ng*t/ml). Additionally, bioavailability and absorption efficiency of KPT-335 after oral administration is dependent on feeding, therefore corticosteroid administration to improve appetite in some patients may subsequently promote more consistent plasma drug levels.

### Clinical toxicities

The most common AEs across all dose groups related to KPT-335 included: anorexia (*n* = 27, 45%), weight loss (*n* = 18, 31%), vomiting (*n* = 15, 26%), lethargy (*n* = 10, 17%) and diarrhea (*n* = 7, 12%). Most events were considered grade 1 or 2 (73/77, 95%).

No difference was observed in the AE profile when comparing dosing regimens (Additional file 1: Table S1). Of 9 dogs with serious adverse events, three had events attributed to the drug. These include weight loss, weakness and hepatopathy. Other events including hypercalcemia, respiratory distress and hemogram abnormalities occurred in the setting of disease progression and were unlikely to be related to KPT-335. Additionally, protein losing nephropathy was seen in one dog, and did not resolve after discontinuation of KPT-335.

## Discussion

Canine lymphoma is the most common hematopoietic neoplasm diagnosed in dogs [13, 14]. Multi-agent protocols used to treat canine lymphoma typically consist of a combination of prednisone, vincristine, cyclophosphamide and doxorubicin, with response rates > 80% and median survival times of 10–14 months reported [15]. Despite continued research efforts to improve upon patient outcome, the prognosis for canine lymphoma has remained unchanged for over 30 years. Therefore, leveraging novel therapeutic targets is necessary to improve outcomes in patients.

The oral compound, KPT-335, is a selective inhibitor of nuclear export (SINE). Many SINE compounds have been shown to inhibit cell growth and induce apoptosis in human and canine cancer cell lines including pancreatic, melanoma and mammary carcinomas [4, 16–19]. A phase I study of KPT-330 in humans with solid tumors reported stable disease in multiple tumor types including colorectal carcinoma, squamous cell carcinoma, prostatic carcinoma, melanoma and sarcomas [6]. Recently, KPT-335 was evaluated in dogs with cancer, and a dose of 1.5 mg/kg given three times weekly was tolerated and found to have a clinical benefit in dogs with lymphoma [11]. The most common adverse event associated with KPT-335 therapy in dogs was low grade anorexia. Given that the oral bioavailability of KPT-335 is significantly impacted by feeding, maintaining an appetite was important, and prednisone administration at a low dose was previously found to be effective in treating anorexia [11]. This study sought to expand upon the phase I clinical trial results to evaluate the biologic activity and tolerability of KPT-335 in naïve and relapsed canine NHL.

In the current study, KPT-335 was administered orally in three dose groups. KPT-335 was well tolerated across all dose groups with minimal SAEs reported. Consistent with prior publications, the most common AE was grade 1–2 anorexia, with only one dog that experienced severe (grade 3 or 4) anorexia. One dog experienced protein losing nephropathy which was suspected to be related to drug administration and resolved following discontinuation. Hepatopathy was also seen in one dog, and hepatopathies were also documented in the phase I study in several dogs dosed with KPT-335 at 1.5 mg/kg and 2.0 mg/kg [11]. Adverse events typically associated with cytotoxic chemotherapy were seen with this study (anorexia, vomiting, diarrhea), indicating combination therapy with KPT-335 and conventional chemotherapeutic agents may be challenging with agents that have similar adverse event profiles. A single study looking at the combination of KPT-330 with fludarabine and cytarabine in patients with refractory pediatric leukemia found the dose limiting toxicity to be cerebellar toxicity. Dose limiting toxicities previously observed with administration of KPT-330, including weight loss and anorexia, were not seen in this study, supporting further evaluation of standard chemotherapy combined XPO1 inhibitors such as KPT-335 [20].

A limitation of this study includes the variability of concurrent corticosteroid usage in patients. Since study enrollment required disease progression, it is the expectation that these tumors are refractory to corticosteroids, and therefore observed responses were likely due to KPT-335. First relapse and naïve NHL dogs that received corticosteroids after study enrollment to treat drug-associated anorexia received a median dose less than 0.5 mg/kg/day, with therapy initiated an average of 26 days after enrollment. Corticosteroids were initiated in these dogs while in a partial remission or experiencing stable disease, and additional measurable disease response was not noted in these patients, as would be expected if prednisone use was contributing to disease outcome. The prednisone doses used were well below the dose of corticosteroids typically used as anti-neoplastic therapy (1–2 mg/kg/day) and as KPT-335 was administered for several weeks prior to initiation of low-dose corticosteroids, would not be expected to impact disease progression. However, while the longer TTP noted in dogs receiving steroids after enrollment in the clinical trial could reflect inherent variability in sensitivity to KPT-335, a confounding effect of prednisone administration cannot be excluded. Prednisone has been previously shown to abrogate anorexia associated with KPT-335 more effectively than commercially available appetite stimulants at the time this study was completed, and was utilized in the present study for the same purpose. As mild anorexia is a known clinical toxicity of KPT-335, future studies may investigate the utility of novel appetite stimulants (i.e. capromorelin) to limit the confounding effects of oral corticosteroid use in dogs receiving KPT-335.

Consistent with the phase I clinical trial in spontaneous canine cancers, KPT-335 demonstrated biological activity in both relapsed and naïve canine NHL. KPT-330, another SINE compound, has been shown to produce an objective response in patients with refractory or relapsed hematologic malignancies [9, 10]. Taken together, these data support the notion that KPT-335 is effective in both naïve and relapsed lymphoma.

The overall median TTP was 43 and 24 days, which is consistent with expected single agent non-cytotoxic therapy for naïve and first relapse canine lymphoma [15]. Forty percent of patients remained on the study for at least 8 weeks, suggesting a substantial proportion of canine lymphoma patients have a significant, sustained benefit from KPT-335 treatment. The durability of KPT-330 as a single agent cancer therapy has also been demonstrated in refractory/relapsed acute myeloid leukemia patients by lengthening median progression-free survival [9]. Single agent targeted therapies can be associated with favorable initial responses, however combination therapy is often necessary to achieve sustained clinical responses. Combination therapy with XPO1 inhibitors, especially KPT-330, and conventional chemotherapeutic agents has shown promising synergistic effects in a variety of human tumor types [21, 22]. In light of previous studies with SINE compounds in human and canine cancers, the findings of this study may provide opportunities for combined therapy with KPT-335 and cytotoxic agents in human and canine patients that have developed resistance to standard chemotherapeutic agents and protocols across a variety of tumor types.

## Conclusions

In summary, this phase II study illustrates the effectiveness and general safety of KPT-335 as a single agent therapy against naïve and first relapse canine lymphoma. Potential future directions include use of combining KPT-335 with cytotoxic chemotherapeutic agents in canine lymphoma.

## Additional file

Additional file 1: **Table S1.** Treatment emergent adverse events. (DOCX 19 kb)

## Abbreviations

AE: Adverse event
AML: Acute myeloid leukemia
AUC: Area under the curve
Cmax: Peak concentration
CR: Complete response
DCR: Disease control rate
DOR: Duration of response
GRP: Growth regulatory proteins
NHL: Non-Hodgkin’s lymphoma
ORR: Objective response rate
PK: Pharmacokinetics
PR: Partial response
SAE: Serious adverse event
SD: Stable disease
SINE: Selective inhibitor of nuclear export
Tmax: Time of observed maximum concentration
TSP: Tumor suppressor proteins
TTP: Time to progression
XPO1: Exportin-1
