# Supplementary material for: Phase II study of the oral selective inhibitor of nuclear export (SINE) KPT-335 (verdinexor) in dogs with lymphoma
Source: BMC Vet Res. 2018 Aug 24;14:250. doi: 10.1186/s12917-018-1587-9 (PMC6109271; doi:10.1186/s12917-018-1587-9)
Supplement: Supplementary file 1 — Table S1. Treatment emergent adverse events. (DOCX 19 kb) [file 12917_2018_1587_MOESM1_ESM.docx]

Supplemental Table 1: Treatment emergent adverse events.

| **Standard Term** | | **1.5 mg/kg PO 3 times weekly** | **1.25 mg/kg PO 3 times weekly** | **1.25 mg/kg twice weekly for 2 weeks, then 1.5 mg/kg twice weekly** | **Total** |
| --- | --- | --- | --- | --- | --- |
|  | | **n=13**  **n (%), E** | **n=35**  **n (%), E** | **n=10**  **n (%), E** | **n=58**  **n (%), E** |
| Any AE | | 13 (100.0%),19 | 35 (100.0%), 114 | 10 (100.0%), 42 | 58 (100.0%), 175 |
|  | |  |  |  |  |
| Anorexia | All | 6 (54.5%), 8 | 16 (45.7%), 23 | 5 (50.0%), 6 | 27 (46.6%), 37 |
|  | severe | 0 (0.0%), 0 | 1 (2.9%), 1 | 0 (0.0%), 0 | 1 (1.7%), 1 |
| Vomiting | All | 2 (15.4%), 2 | 11 (31.4%), 18 | 2 (20.0%), 7 | 15 (25.9%), 27 |
|  | severe | 0 (0.0%), 0 | 0 (0.0%), 0 | 0 (0.0%), 0 | 0 (0.0%), 0 |
| Elevated ALP | All | 0 (0.0%), 0 | 0 (0.0%), 0 | 2 (20.0%), 5 | 2 (3.4%), 5 |
|  | severe | 0 (0.0%), 0 | 0 (0.0%), 0 | 2 (20.0%), 2 | 2 (3.4%), 2 |
| Diarrhea | All | 1 (7.7%), 1 | 4 (11.4%), 4 | 2 (20.0%), 3 | 7 (12.1%), 8 |
|  | severe | 0 (0.0%), 0 | 0 (0.0%), 0 | 0 (0.0%), 0 | 0 (0.0%), 0 |
| Weight loss | All | 2 (15.4%), 3 | 14 (40.0%), 21 | 2 (20.0%), 3 | 18 (31.0%), 27 |
|  | severe | 1 (7.7%), 1 | 0 (0.0%), 0 | 0 (0.0%), 0 | 1 (1.7%), 1 |
| Elevated ALT | All | 0 (0.0%), 0 | 1 (2.9%), 2 | 2 (20.0%), 6 | 3 (5.2%), 8 |
|  | severe | 0 (0.0%), 0 | 0 (0.0%), 0 | 2 (20.0%), 2 | 2 (3.4%), 2 |
| Lethargy | All | 0 (0.0%), 0 | 9 (25.7%), 12 | 1 (10.0%), 1 | 10 (17.2%), 13 |
|  | severe | 0 (0.0%), 0 | 0 (0.0%), 0 | 0 (0.0%), 0 | 0 (0.0%), 0 |
| Anemia | All | 0 (0.0%), 0 | 1 (2.9%), 1 | 0 (0.0%), 0 | 1 (1.7%), 1 |
|  | severe | 0 (0.0%), 0 | 0 (0.0%), 0 | 0 (0.0%), 0 | 0 (0.0%), 0 |
| Elevated BUN | All | 0 (0.0%), 0 | 2 (5.7%), 2 | 0 (0.0%), 0 | 2 (3.4%), 2 |
|  | severe | 0 (0.0%), 0 | 0 (0.0%), 0 | 0 (0.0%), 0 | 0 (0.0%) ,0 |
| Polydipsia | All | 1 (7.7%), 1 | 4 (11.4%), 4 | 0 (0.0%), 0 | 5 (8.6%), 5 |
|  | severe | 0 (0.0%), 0 | 0 (0.0%), 0 | 0 (0.0%), 0 | 0 (0.0%), 0 |
| Thrombocytopenia | All | 0 (0.0%), 0 | 3 (23.1%), 4 | 1 (10.0%), 1 | 4 (6.9%), 5 |
|  | severe | 0 (0.0%), 0 | 0 (0.0%), 0 | 0 (0.0%), 0 | 0 (0.0%), 0 |
| Polyuria | All | 1 (7.7%), 1 | 3 (8.6%), 3 | 0 (0.0%), 0 | 4 (6.9%), 4 |
|  | severe | 0 (0.0%), 0 | 0 (0.0%), 0 | 0 (0.0%), 0 | 0 (0.0%), 0 |
| Leukopenia | All | 0 (0.0%), 0 | 3 (8.6%), 3 | 1 (10.0%), 1 | 4 (6.9%), 4 |
|  | severe | 0 (0.0%), 0 | 0 (0.0%), 0 | 0 (0.0%), 0 | 0 (0.0%), 0 |
| Proteinuria | All | 0 (0.0%), 0 | 1 (2.9%), 1 | 0 (0.0%), 0 | 1 (1.7%), 1 |
|  | severe | 0 (0.0%), 0 | 0 (0.0%), 0 | 0 (0.0%), 0 | 0 (0.0%), 0 |
| Urinary tract infection | All | 0 (0.0%), 0 | 1 (2.9%), 1 | 0 (0.0%), 0 | 1 (1.7%), 1 |
|  | severe | 0 (0.0%), 0 | 0 (0.0%), 0 | 0 (0.0%), 0 | 0 (0.0%), 0 |
| Elevated AST | All | 0 (0.0%), 0 | 0 (0.0%), 0 | 1 (10.0%), 1 | 1 (1.7%), 1 |
|  | severe | 0 (0.0%), 0 | 0 (0.0%), 0 | 0 (0.0%), 0 | 0 (0.0%), 0 |
| Cough | All | 0 (0.0%), 0 | 1 (2.9%), 1 | 0 (0.0%), 0 | 1 (1.7%), 1 |
|  | severe | 0 (0.0%), 0 | 0 (0.0%), 0 | 0 (0.0%), 0 | 0 (0.0%), 0 |
| Nausea | All | 0 (0.0%), 0 | 1 (2.9%), 2 | 1 (10.0%), 1 | 2 (3.4%), 3 |
|  | severe | 0 (0.0%), 0 | 0 (0.0%), 0 | 0 (0.0%),0 | 0 (0.0%), 0 |
| Hypoproteinemia | All | 0 (0.0%), 0 | 1 (2.9%), 1 | 0 (0.0%),0 | 1 (1.7%), 1 |
|  | severe | 0 (0.0%), 0 | 0 (0.0%), 0 | 0 (0.0%),0 | 0 (0.0%), 0 |
| Dyspnea | All | 0 (0.0%), 0 | 0 (0.0%), 0 | 1 (10.0%), 1 | 1 (1.7%), 1 |
|  | severe | 0 (0.0%), 0 | 0 (0.0%), 0 | 0 (0.0%),0 | 0 (0.0%), 0 |
| Elevated GGT | All | 0 (0.0%), 0 | 0 (0.0%), 0 | 1 (10.0%),2 | 1 (1.7%), 2 |
|  | severe | 0 (0.0%), 0 | 0 (0.0%), 0 | 0 (0.0%),0 | 0 (0.0%), 0 |
| Hypoalbuminemia | All | 0 (0.0%), 0 | 1 (2.9%), 1 | 0 (0.0%),0 | 1 (1.7%), 1 |
|  | severe | 0 (0.0%), 0 | 0 (0.0%), 0 | 0 (0.0%),0 | 0 (0.0%), 0 |
| Isosthenuria | All | 0 (0.0%), 0 | 2 (5.7%), 2 | 0 (0.0%),0 | 2 (3.4%), 2 |
|  | severe | 0 (0.0%), 0 | 0 (0.0%), 0 | 0 (0.0%),0 | 0 (0.0%), 0 |
| Pyoderma | All | 0 (0.0%), 0 | 1 (2.9%), 1 | 0 (0.0%),0 | 1 (1.7%), 1 |
|  | severe | 0 (0.0%), 0 | 0 (0.0%), 0 | 0 (0.0%),0 | 0 (0.0%), 0 |
| Edema | All | 0 (0.0%), 0 | 1 (2.9%), 1 | 0 (0.0%),0 | 1 (1.7%), 1 |
|  | severe | 0 (0.0%), 0 | 0 (0.0%), 0 | 0 (0.0%),0 | 0 (0.0%), 0 |
| Cachexia | All | 0 (0.0%), 0 | 2 (5.7%), 2 | 0 (0.0%),0 | 2 (3.4%), 2 |
|  | severe | 0 (0.0%), 0 | 0 (0.0%), 0 | 0 (0.0%),0 | 0 (0.0%), 0 |
| Thin | All | 1 (7.7%), 1 | 0 (0.0%), 0 | 0 (0.0%),0 | 1 (1.7%), 1 |
|  | severe | 0 (0.0%), 0 | 0 (0.0%), 0 | 0 (0.0%),0 | 0 (0.0%), 0 |
| Lymphadenitis | All | 0 (0.0%), 0 | 1 (2.9%), 1 | 0 (0.0%),0 | 1 (1.7%), 1 |
|  | severe | 0 (0.0%), 0 | 0 (0.0%), 0 | 0 (0.0%),0 | 0 (0.0%), 0 |
| Obtunded | All | 1 (7.7%), 1 | 0 (0.0%), 0 | 0 (0.0%),0 | 1 (1.7%), 1 |
|  | severe | 0 (0.0%), 0 | 0 (0.0%), 0 | 0 (0.0%),0 | 0 (0.0%), 0 |
| Protein losing nephropathy | All | 0 (0.0%), 0 | 1 (2.9%), 1 | 0 (0.0%),0 | 1 (1.7%), 1 |
|  | severe | 0 (0.0%), 0 | 1 (2.9%), 1 | 0 (0.0%),0 | 1 (1.7%), 1 |

E = number of events
